# Reactivation of Coccidioidomycosis in a Mouse Model of Asymptomatic Controlled Disease

**DOI:** 10.3390/jof8100991

**Published:** 2022-09-21

**Authors:** Lisa F. Shubitz, Daniel A. Powell, Sharon M. Dial, Christine D. Butkiewicz, Hien T. Trinh, Amy P. Hsu, Adam Buntzman, Jeffrey A. Frelinger, John N. Galgiani

**Affiliations:** 1 Valley Fever Center for Excellence, University of Arizona, 1656 E Mabel St., Tucson, AZ 85719, USA; 2 Department of Immunobiology, University of Arizona, 1656 E Mabel St., Tucson, AZ 85724, USA; 3 College of Veterinary Medicine, University of Arizona, 1580 E Hanley Blvd, Oro Valley, AZ 85737, USA; 4 Laboratory of Clinical Immunology and Microbiology, NIAID, Building 10, 10 Center Drive, Bethesda, MD 20892, USA; 5 BIO5, University of Arizona, 1657 E Helen St., Tucson, AZ 85719, USA; 6 Department of Medicine, University of Arizona, 1501 N. Campbell Ave, Tucson, AZ 85724, USA

**Keywords:** *Coccidioides*, mice, immunosuppression, reactivation, granuloma

## Abstract

The majority of human coccidioidomycosis infections are asymptomatic or self-limited but may have sequestered spherules in highly structured granulomas. Under immunosuppression, reactivation of fungal growth can result in severe disease. B6D2F1 mice asymptomatically infected with *C. posadasii* strain 1038 were immunosuppressed with dexamethasone (DXM) in drinking water. Treated mice died 16–25 days later, while untreated mice survived (*p* < 0.001). Flow cytometry of lung granulomas on days 5, 10, 15, and 20 of DXM treatment showed immune cell populations decreased 0.5–1 log compared with untreated mice though neutrophils and CD19^+^IgD^−^IgM^−^ cells rebounded by day 20. Histopathology demonstrated loss of granuloma structure by day 5 and increasing spherules through day 20. On day 20, T-cells were nearly absent and disorganized pyogranulomatous lesions included sheets of plasma cells and innumerable spherules. Mice given DXM for 14 days then stopped (DXM stop) survived 6 weeks (9/10). Lung fungal burdens were significantly lower (*p* = 0.0447) than mice that continued treatment (DXM cont) but higher than untreated mice. Histopathologically, DXM stop mice did not redevelop controlled granulomas by sacrifice, though T-cells were densely scattered throughout the lesions. This demonstrates a mouse model suitable for further study to understand the immunologic components responsible for maintenance control of coccidioidomycosis.

## 1. Introduction

The majority of human infections with *Coccidioides posadasii* or *C. immitis* fungi are asymptomatic or self-limited, and patients recover completely [1]. However, the fungus is not necessarily eradicated and may continue to survive sequestered in mature granulomas [2,3]. Experiences with AIDS, solid organ transplantation, and other immunosuppressive medical therapies show that these quiescent granulomas can be perturbed to the point that the patient develops severe, symptomatic coccidioidomycosis [4,5,6,7,8].

Until recently, there was no mouse model to explore the host requirements for maintaining remission. We recently reported that C57BL/6 mice infected with *C. posadasii* strain 1038 (Cp1038) develop a slowly progressive but ultimately lethal infection, while C57BL/6 × DBA/2 F1 (B6D2F1) mice develop stable, asymptomatic disease characterized by one or a few mature lung granulomas containing a low number of spherules and minimal dissemination [9]. In that report, B6D2F1 mice developed stable total lung fungal burdens of 1–5 × 10^4^ colony-forming units (cfu) by 4 weeks post-infection through 16 weeks. Subsequently, we infected B6D2F1 mice and followed them for a total of 34 weeks; all animals remained healthy, and their mean lung fungal burden was 1.58 × 10^3^, about 1 log lower than 16-week mice from the previous studies, and three of five spleens had no growth (range, 0–30 cfu/spleen). This demonstrates that control of the infection can be protracted without eradication in this mouse strain. In the current report, we show that treatment with a broad immunosuppressant, dexamethasone (DXM), disrupts the architecture of stable granulomas. Histopathological changes in the granuloma and fungal burden increases are demonstrated and supported by flow cytometric analysis of granuloma cell populations in mice treated with DXM compared with untreated controls. These findings provide a model in which the immunologic control of chronic coccidioidal infection in B6D2F1 mice can be dissected.

## 2. Materials and Methods

### 2.1. Mice

(C57BL/6J x DBA/2J)F1 (stock number 100006) were purchased from Jackson Laboratories (Bar Harbor, ME, USA) at 7–8 weeks of age. Female mice were group housed and cared for according to PHS guidelines at animal biosafety level 3. Animals were monitored daily for health and activity. Mice with progressive disease were euthanized humanely when weight loss was ≥30% and/or mice were lethargic, isolated from cagemates, demonstrated weakness, were ≥10% dehydrated by skin turgor test, or had any CNS signs or difficulty ambulating and obtaining food and water. All procedures were approved by the Institutional Animal Care and Use Committee of the University of Arizona.

### 2.2. Arthroconidia

*C. posadasii*, strain 1038 (Cp1038), mycelial cultures were grown from a seed bank stored at −80 °C. Cultures were grown on 2X GYE (2% glucose, 2% yeast extract, and 1% agarose) at 30 °C in a BSL3 laboratory (Keating, University of Arizona) for 8 weeks, until mycelia appeared mature. Arthroconidia were prepared for infection as previously described [9,10]. Briefly, the arthroconidia were harvested using the spin bar method in water, filtered through Thermolam^®^ to remove mycelial fragments, and washed twice with centrifugation. Arthroconidia were stored in water in concentrations >1 × 10^7^/mL at 4 °C until use. Counts were made with a hemacytometer, and viability was determined by enumerating 10-fold serial dilutions grown on GYE at 37 °C for 96 h. All growth and handling of live Cp1038 cultures were performed at biosafety level 3.

### 2.3. Mouse Infection and Immunosuppression

Mice were anesthetized with a cocktail of ketamine and xylazine (80 mg/kg ketamine, 8 mg/kg xylazine) administered intraperitoneally. Mice were infected by intranasal insufflation with a target dose of 50 Cp1038 arthroconidia in sterile saline for injection. DXM was administered in drinking water (6 mg/L) [11] starting 4–6 weeks after infection. Mice were either treated for 14 days then changed back to plain water, or were treated continuously until timed euthanasia or moribundity (described above). Controls received plain water throughout.

### 2.4. Histopathology

Lungs were divided with the left lung, which consists of a single, unsegmented lobe, fixed in 4% paraformaldehyde (PFA) for staining. Tissues were embedded in paraffin, and 5 µm thick sections were stained routinely with hematoxylin and eosin (H&E) for each mouse. Additional special stains were applied to left lungs with observed lesions. Spherules were visualized by staining with a *Coccidioides*-specific polyclonal goat anti-PRA antibody as previously reported [12] or a periodic acid Schiff (PAS) stain [13]. Immunohistochemical stains included anti-CD3 for T-cells (clone SP7, rabbit monoclonal, Abcam, Waltham, MA, USA), anti-CD138 for mature plasma cells (clone 281–2, Biotin anti-mouse, Biolegend, San Diego, CA, USA), and NIMP-R14 for neutrophils (rabbit monoclonal, Abcam).

### 2.5. Organ Fungal Burden

Either the entire lung (8 mice) or the right lung plus accessory lobe (10 mice) and spleens (18 mice) were homogenized in 1 mL of sterile, isotonic saline using a rotary homogenizer (Glas-Col, Terra Haute, Indiana). Ten-fold serial dilutions were plated on GYE and incubated for 96 h at 37 °C to enumerate colonies. Fungal burden is reported as colony-forming units (cfu) per organ (entire lung, right lung, or spleen).

### 2.6. Flow Cytometric Characterization of Granulomas

Right lungs, including the accessory lobe, of treated and control mice sacrificed at baseline and on days 5, 10, 15, and 20 after starting DXM in drinking water were dissected onto a flat surface. Granulomas were closely dissected from lungs using a 3 mm skin biopsy punch to exclude grossly normal lung tissue. Granulomas from each mouse were pooled per mouse into cold RPMI. The granulomas were mechanically filtered over a 70 µm cell strainer to prepare single-cell suspensions. Red blood cells were lysed with ACK buffer, then cells were washed, resuspended, and enumerated via Vi-Cell (Beckman Coulter) to obtain viable counts. Cells were incubated for 30 min at 4 °C with 2.4G2 supernatant to block Fc receptors, then stained with labeled antibodies for 30 min at 4 °C in the dark. Antibodies are shown in Table S1. After washing three times with PBS + 2% BSA, cells were fixed with 4% paraformaldehyde and analyzed on an LSRII flow cytometer (BD Biosciences). The data were analyzed with FlowJo software (Ashland, OR, USA). Total cell numbers were calculated using viable counts determined by Vi-Cell using Trypan Blue exclusion. Gates were set based on fluorescence minus one on normal spleens and applied across all samples.

### 2.7. Statistical Analysis

Statistical analysis was performed in GraphPad Prism version 9.4.0 (San Diego, CA, USA). Data were assessed for normality by Shapiro–Wilk and then log transformed. Statistical comparisons were made using Mann–Whitney U or Kruskal–Wallis as appropriate. *p* values were corrected for multiple comparisons, and results were considered significant at *p* < 0.05.

## 3. Results

### 3.1. Fungal Growth, Dissemination, and Mouse Mortality Occur following Administration of DXM

**Figure 1:**
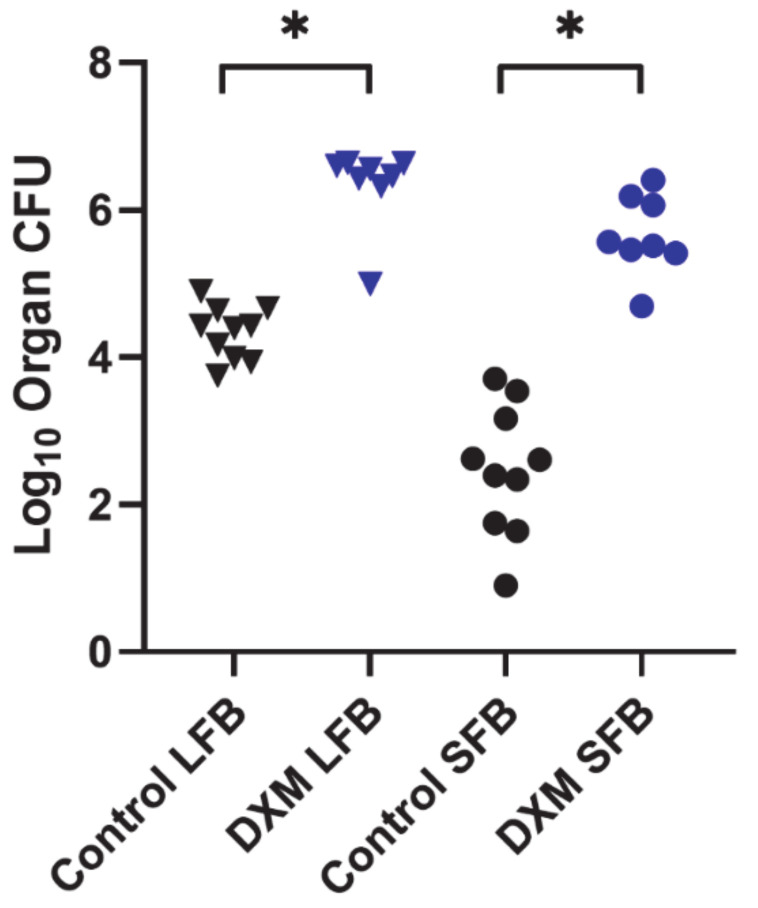
Organ fungal burdens of DXM treated and untreated mice. B6D2F1 mice (n = 8/group) were infected with 50 spores of Cp1038, and lung and spleen fungal burdens quantitated at termination after treatment with DXM in drinking water or no treatment. Fungal burdens were significantly higher in both lungs (LFB) and spleens (SFB) following continuous treatment with DXM compared with the untreated controls (*p* < 0.0001, both comparisons). (* Indicates statistical significance; statistical analysis—Kruskal–Wallis.).

Two studies were performed (N = 8/grp and N = 10/grp) in which stably infected mice were administered DXM, and fungal burdens were measured approximately 6 weeks later. In one study, a group of eight mice began DXM treatment on post-infection day 42 and was compared with an untreated infected control group. DXM-treated mice became moribund and were sacrificed between days 58 and 67 post-infection (16–25 days after starting DXM), and all controls survived until scheduled sacrifice 105 days post-infection (*p* = 0.0001). In addition to reduced survival, lung and spleen fungal burdens of treated mice were significantly increased compared with the controls (*p* < 0.0001, both comparisons) (Figure 1).

**Figure 2:**
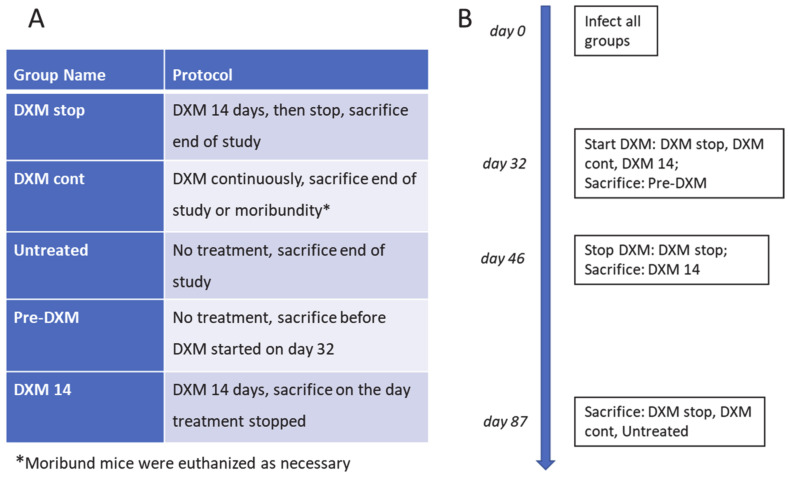
Treatment and sacrifice protocol for comparison of mice treated for two weeks or continuously with DXM, 6 mg/L, in drinking water. Groups and treatment conditions (**A**) and timeline of study (**B**) for 2-week and continuous administration of DXM with right lung fungal burden and left lung histopathology.

**Figure 3:**
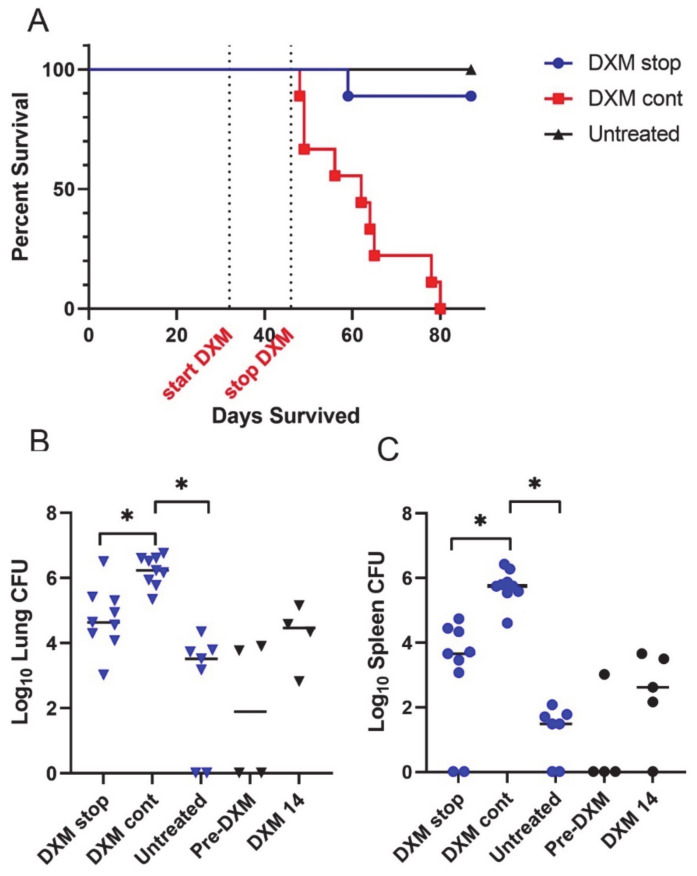
Survival and fungal burdens of 2-week or continuous treatment with DXM. (**A**) Mice (n = 10/group) treated continuously with DXM (DXM cont) became moribund before scheduled sacrifice (day 87 post-infection). (**B**) Right lung fungal burdens of mice treated with DXM for 14 days and then discontinued (DXM stop) were intermediate compared with mice treated continuously (DXM cont) and untreated mice. DXM cont fungal burdens were significantly higher than the untreated (*p* < 0.0001) and DXM stop mice (*p* = 0.0447). The right lung fungal burdens of mice before the start of DXM (pre-DXM) and on the 14th day of treatment (DXM 14) show that the fungal burden increased rapidly with immunosuppression and remained approximately the same 6 weeks after discontinuation of DXM. (**C**) Spleen fungal burdens paralleled lung data (DXM stop vs. DXM cont, *p* = 0.0105; untreated vs. DXM cont, *p* = 0.0001). (* Indicates statistical significance. Statistical analysis: survival—Mann–Whitney U; organ fungal burdens—Kruskal–Wallis).

In a second study (Figure 2), we assessed how 2 weeks of DXM administration differed from continuous treatment or no treatment through post-infection day 87. Starting post-infection day 32, 10 mice per group were treated with DXM for 14 days (DXM stop) or continuously (DXM cont) or were left untreated (Untreated). To enrich the assessment, five additional mice were sacrificed on day 32 before DXM began (Pre-DXM) and five treated with DXM were sacrificed on day 46 after 14 days of DXM (DXM 14). At sacrifice, right plus accessory lung lobes and spleens were quantitatively cultured. Figure 3A shows that 9/10 DXM stop and all untreated mice survived, while DXM cont mice became moribund as in the previous study. The DXM cont mice had a >2 log increase in lung fungal burdens vs. untreated (*p* = 0.0001). The DXM stop mice were in between, about 1 log higher than untreated mice and 1 log lower than DXM cont mice (*p* = 0.1896 and *p* = 0.0447, respectively) (Figure 3B). Spleen fungal burden comparisons were similar in scope of differences and significance and verify the observation from the first study that dissemination occurs rapidly with severe immunosuppression (Figure 3C).

In both studies, the continuous administration of DXM resulted in a ≥100-fold increase in lung and spleen fungal burdens with significantly shortened survival. In particular, dissemination drastically worsened as evidenced by the increase in spleen fungal burdens and observation of miliary lesions in the livers and spleens of the DXM-treated mice. Visible abdominal lesions were absent or rare in untreated controls. Mice in which the immunosuppression was stopped after two weeks were intermediate in their organ fungal burdens but demonstrated significant dissemination.

### 3.2. All Immune Cell Populations Decreased following DXM Treatment

**Figure 4:**
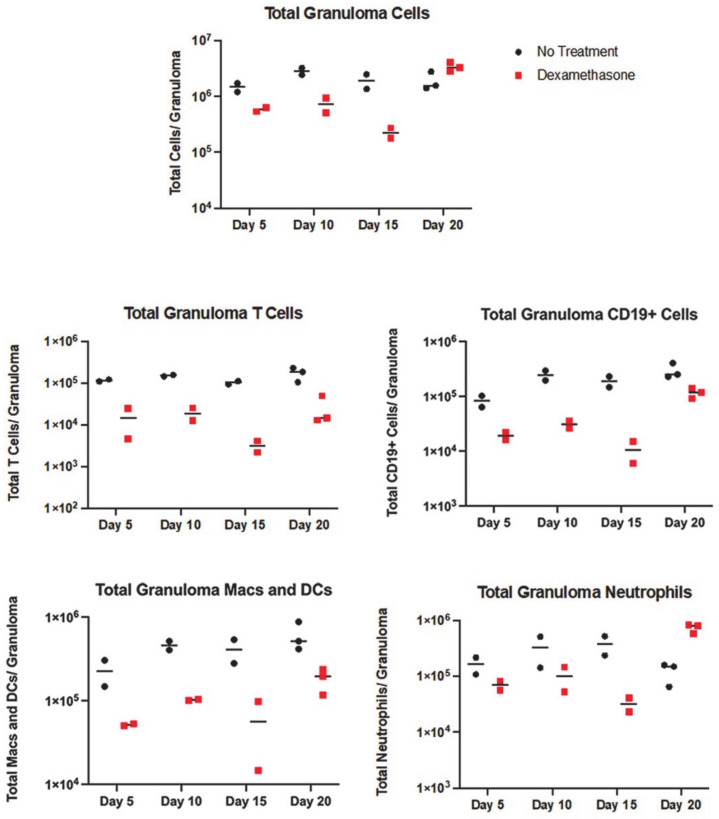
Flow cytometry counts of immune cells in treated and untreated mice. Total immune cells in the granulomas reduce between day 5 and 15 after starting DXM. There is a rebound on day 20. T-cell populations remain depressed at all time points, along with the modest reduction in macrophage/DC lines, but CD19^+^ B-lineage cells and neutrophils show an increase on day 20. Population subsets are shown in Supplementary Figures S1 and S2.

DXM-treated and control mice (N = 2–3 mice per time point) were sacrificed 5, 10, 15, or 20 days after beginning DXM, with two mice sacrificed on day 0 as a technical control, and cell populations of the right lung granulomas were quantitated by flow cytometry. Total viable cell counts were measured, then a panel of stains (Table S1) applied to determine the composition of the immune cells in the granulomas. Total viable cells rapidly decreased approximately 10-fold in the granulomas by days 10 and 15. A rebound in the total cell number was seen on day 20 (Figure 4). All lymphocytes, including all subpopulations measured, decreased about 10-fold (Figure 4, Figures S1 and S2A). In contrast to T-cells, CD19^+^IgD^−^IgM^−^ B lineage cells (likely plasma cells, see below) increased by day 20, while naïve and maturing (CD19^+^IgD^+^IgM^+^, CD19^+^IgD^+^IgM^−)^ B lineage cell numbers remained depressed (Figure 3 and Figure S2A). Macrophages and DCs showed a less radical decline but also remained decreased compared with controls, except for a rebound in DCs to near starting levels on day 20 (Figure S2B). Neutrophils were also suppressed through day 15, but this population increased above the control numbers by day 20. The increase in neutrophils and CD19^+^IgD^-^IgM^-^ B lineage cells can account for the total cell count recovery on day 20. Although neutrophil responses to exogenous glucocorticoids are complicated [14], it is plausible that the initial reduction in neutrophils in the granulomas are, in part, the result of their normal short lifespan and reduced extravasation from the DXM. Even though DXM was still being given, we speculate that the large increase in neutrophils in the day 20 lesions stems from chemotaxis related to the hugely increased numbers of spherules and endospores [15].

### 3.3. Granulomas Break down with DXM Treatment

**Figure 5:**
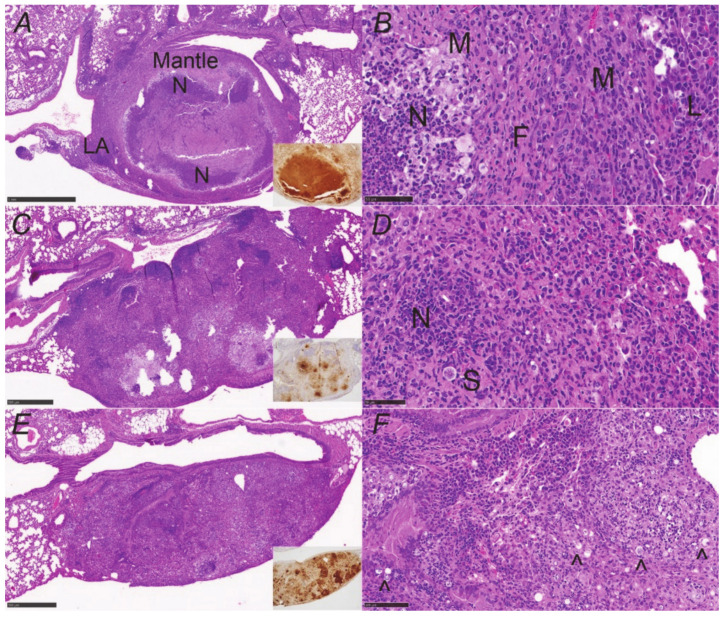
Granuloma destruction over time with DXM suppression. (**A**) Structure of a controlled granuloma showing it is well-demarcated from the surrounding lung and consists of a necrotic center with many neutrophils (N) and a mantle region composed of macrophages, fibroblasts, scattered neutrophils, and scattered lymphocytes (inset, neutrophil stain). Lymphoid aggregates (LA) are common on the periphery of the margin. (**B**) Higher-power image of (**A**) highlighting layers; F = fibroblasts, M = macrophages, L = lymphocytes, and N = neutrophils. (**C**) Day 5 of DXM administration shows a huge reduction in neutrophils in the core of the lesion (inset, neutrophil stain) and (**D**) is a higher-power image from the right side of the periphery with a large spherule (S) and a cluster of neutrophils (N) near the margins of the lesion. (**E**) Day 20 lesion showing complete loss of granuloma structure characterized by mixed inflammation of macrophages and resurgent neutrophils (inset, neutrophil stain) (pyogranulomatous lesion) with innumerable spherules throughout. (**F**) High-power image of (**E**) with some of the abundant spherules marked (black carat). Sheets of plasma cells are the main lymphocytic cells present. (H&E stain; insets—NIMP-R14 neutrophil stain. Magnification: (**A**,**C**,**E**)—×40; (**B**,**D**,**F**)—×200).

Figure 5A,B shows an example of the structure of a stable (controlled) lung granuloma in B6D2F1 mice prior to immunosuppression. Generally, the lesion is well-demarcated from normal lung and has a necrotic center with low to moderate spherule numbers and many neutrophils. Surrounding this is a fibrogranulomatous mantle region composed of macrophages and fibrocytes with scattered lymphocytes and neutrophils; there are lymphoid aggregates on the periphery of the mantle, which we have previously reported to be composed of mixed lymphocytes [12]. This very organized structure effectively walls off the infection.

**Figure 6:**
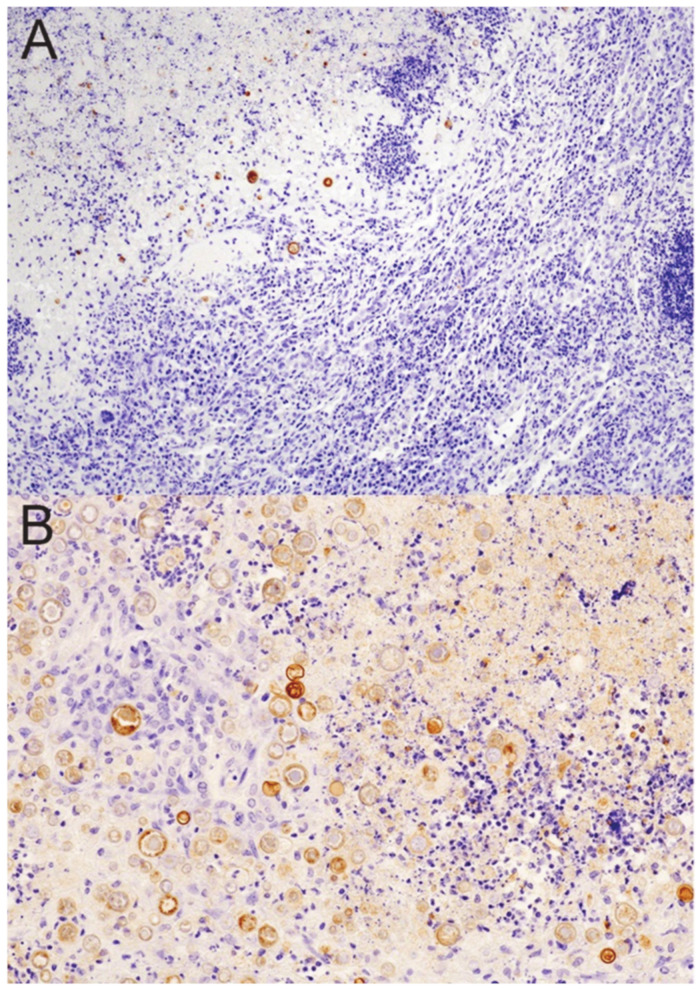
*Coccidioides*-specific stain of spherules prior to and after DXM immunosuppression. (**A**) There are few spherules (brown stain) confined to the necrotic core of a controlled granuloma in an untreated mouse. (**B**) Sheets of spherules are found throughout the disorganized pyogranulomatous lesion (former granuloma) in a mouse treated for 20 days with DXM. Note that many of the spherules in this image are only minimally stained. (Stain: polyclonal goat anti-PRA Ab with a hematoxylin counterstain; magnification (**A**)—×100; (**B**)—×200).

With the administration of immunosuppressive doses of DXM, granulomas underwent significant changes by day 5 (Figure 5C). The neutrophils nearly disappeared from the lesions (Figure 5C inset) and lymphocytes were visibly reduced, though lymphoid aggregates could still be seen on lesion margins. Spherules were increased in both the necrotic core of the lesion and throughout the mantle, moving peripherally toward the lesion edges (Figure 5D). It is difficult to visualize the decrease in macrophages documented in the flow cytometric evaluation on day 5 because they dominate the day 5 lesions in immunosuppressed mice. Day 10 and 15 slides showed further decreases in lymphocytes and loss of lymphoid aggregates, rising spherule numbers, and an increase in “foamy” macrophages, while neutrophil numbers remained reduced. In the day 20 lungs, lesions consisted of a dense region of unorganized mixed inflammation characterized by abundant neutrophils and macrophages (pyogranulomatous lesion) where there used to be a granuloma (Figure 5E,F). There were high numbers of spherules throughout, enormously increased from pretreatment (Figure 6).

**Figure 7:**
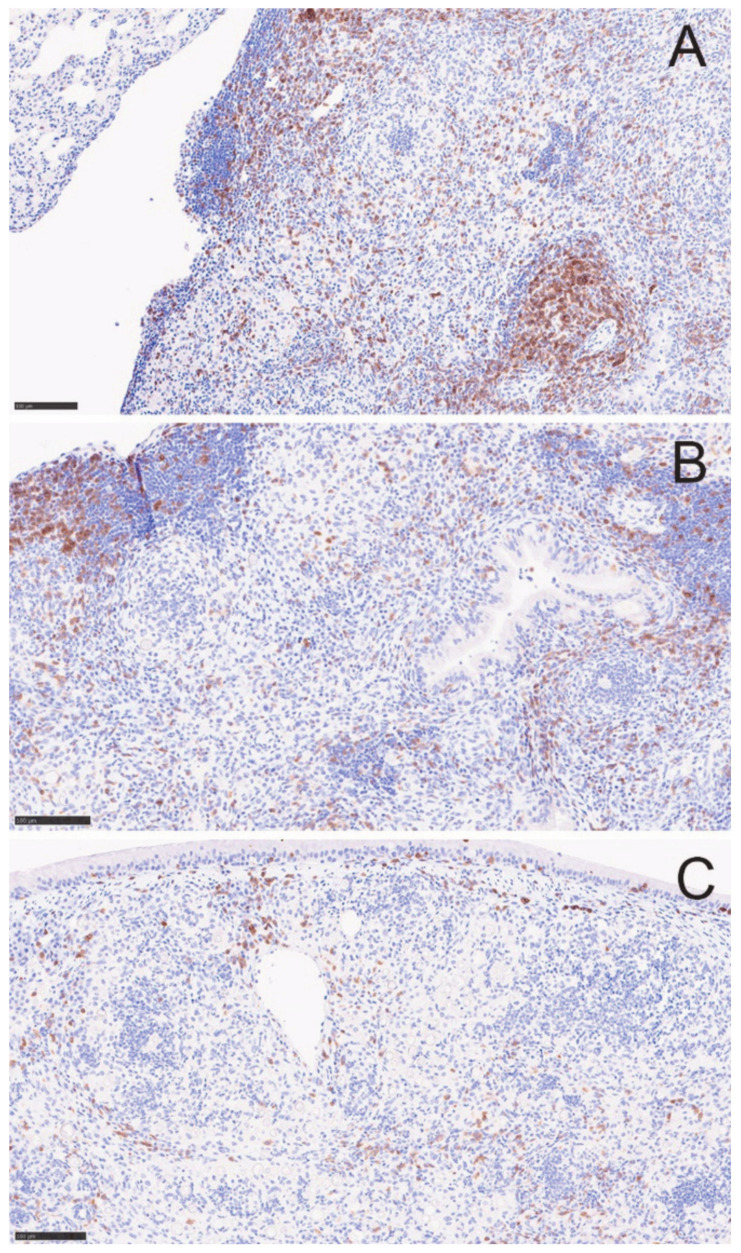
T-cells diminish over 20 days in DXM treated mice. CD3^+^ T-cells diminish in the lesions over time. (**A**) before treatment, (**B**) DXM Day 5, and (**C**) DXM day 20. Images are from the same lesions as in Figure 3 and further demonstrate the loss of the lymphoid aggregates. (Stain—anti-CD3 (brown) with hematoxylin counterstain; magnification ×100).

T-cells decreased by day 5 and appeared even lower on day 20 (Figure 7), correlating with the lack of recovery seen by flow cytometry. However, we observed sheets of plasma cells in the day 20 lesions (H&E stain) that were verified immunohistochemically by staining for CD138 (Figure S3). We think these correspond to the increased population of CD19^+^IgD^−^IgM^−^ B lineage cells on flow cytometric analysis (Figure S4). Plasma cells have been shown to be present in *M. tuberculosis* granulomas in a macaque model as well [16]. Overall, the histological observations in lesions between 5 and 20 days after starting DXM correlated well with the flow cytometry findings, though no statistical analysis can be performed due to the small number of animals.

Since DXM treatment resulted in the loss of granuloma structure, 500-fold higher lung and spleen fungal burdens (dissemination), and mouse mortality within a few weeks of starting administration, we grew interested in what would happen to the granuloma structure and the fungal burden when DXM is given long enough to radically disrupt the controlled granuloma and then stopped. We wanted to know if mice regain control of the infection and redevelop characteristic controlled granulomas with a necrotic center and the mantle region of fibrocytes, macrophages, neutrophils, T-cells, and peripheral lymphoid aggregates. Left lungs from the study described in Figure 2 were fixed, sectioned, and stained.

**Figure 8:**
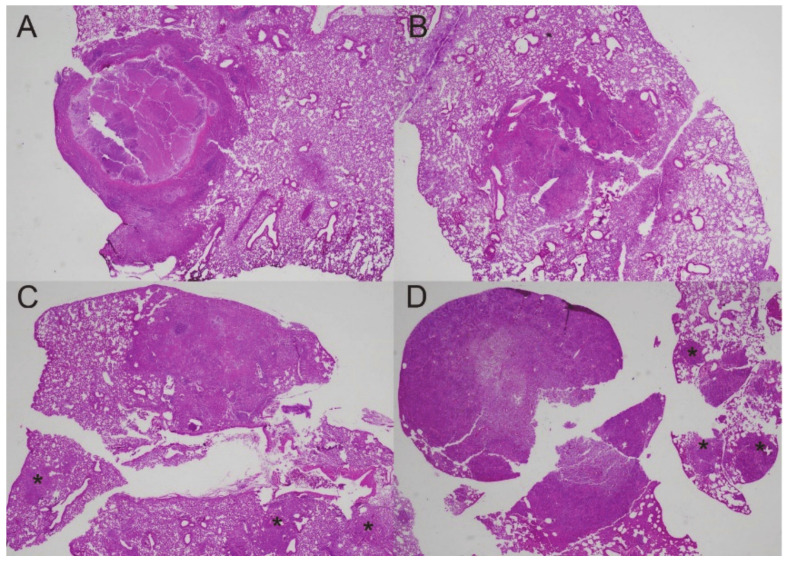
Granulomas at different time points before and after DXM suppression. (**A**) Controlled granuloma in untreated mouse; (**B**) after 14 days of DXM, the structure of the lesion is lost; (**C**) 6 weeks after stopping DXM, the pyogranulomatous lesion has not recovered the structure seen in (**A**), and there are additional centers of pyogranulomatous inflammation in the lung lobe (asterisks); (**D**) in a mouse treated continuously with DXM, the primary lesion on the left side of the image is intensely neutrophilic with innumerable spherules, and there are multiple additional lesions with many spherules beyond the large initial one (asterisks). (Stain—HE; magnification ×20).

**Figure 9:**
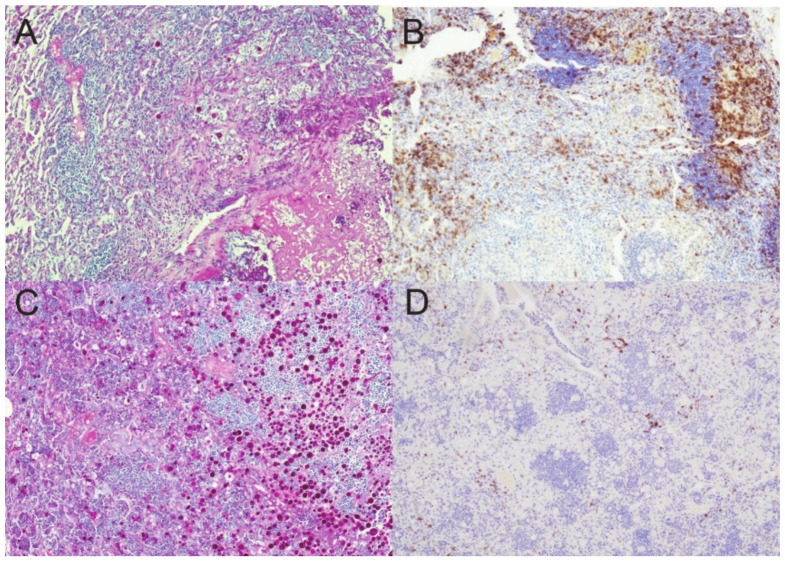
Spherules and T-cells in DXM stop and DXM cont mice. Spherules (**A**) were few and T-cells (**B**) abundant throughout the pyogranulomatous lesions of mice 6 weeks after discontinuing a 14-day course of DXM. By contrast, spherules were numerous (**C**) and T-cells nearly absent (**D**) in mice receiving DXM continuously. (Stain—(**A**,**C**)—periodic acid Schiff stains spherules dark pink; (**B**,**D**)—anti-CD3 IHC stains the T-cells brown; magnification ×100).

Figure 8A shows an example of a controlled granuloma in an untreated mouse. After 14 days of treatment (DXM 14), the granulomas were replaced with disorganized pyogranulomatous lesions as in the previous study (Figure 8B). Discontinuing DXM prevented mortality and arrested the increased fungal burdens but, surprisingly, did not result in reformation of the controlled granuloma 6 weeks later (Figure 8C). Furthermore, those mice formed additional pyogranulomatous lesions in the lungs, which are not seen in the DXM 14 mice but resembled the lungs of mice that received ongoing DXM (DXM cont) (Figure 8C,D). There were more new pyogranulomatous lesions in the DXM cont mice than the DXM stop mice, but the most notable difference between the lesion character of the two groups was the high number of spherules and nearly absent T-cells in the DXM cont group compared with low spherule numbers and densely scattered T-cells in the DXM stop mice (Figure 9). Since no samples were available for flow cytometry in this study, we do not know what T-cell subsets were present in these lesions. The increases in DCs on day 20 from the flow cytometry study, as well as the large number of T-cells throughout the lesions in the DXM stop mice by histopathology, suggest that the mice were trying to redevelop an immune response to the disease but had not successfully achieved the immune control they exhibited after initial infection. Additional studies of the post-DXM lesions will be important to understanding how the host recovers—if it recovers—from the insult of severe immunosuppression.

## 4. Discussion

These observations demonstrate a mouse model to further understand the immunologic components responsible for maintenance of controlled coccidioidomycosis (CM). The majority of people retain this control for life, but the human literature shows that reactivation under immunosuppression is a clinical problem [4,5,6,7,8]. Therefore, dissecting the absolutely essential elements and the coordinated interplay of cells and signals the host uses to maintain this homeostasis with spherules is important to providing better clinical care for patients.

Though the flow cytometric data in these studies were limited, the histopathological observations and the flow cytometric findings correlated very well. This strengthens the validity of the changes witnessed and also shows a path forward to conduct more in-depth analyses of the elements required to maintain control. Other modalities to closely evaluate the interplay of cells in the controlled granulomas, including transcriptional differences at the central and peripheral regions of the mantle, are appropriate follow up explorations based on these data [17,18].

We expected to see that the broad, severe immunosuppression of systemic dexamethasone administration led to the reactivation of CM with progression to death in mice [11,19]. As is commonly reported in human disease [20,21], this was associated with dissemination, measured in these studies by the huge increase in spleen fungal burden. Additionally, we noted that many new pyogranulomatous lesions, characterized by a mixture of neutrophils and macrophages, formed throughout the lungs with immunosuppression compared with the one to four isolated, controlled granulomas seen in untreated mice. What was surprising was that these additional lesions formed in the lungs of the DXM stop mice over the ensuing six weeks after the immunosuppression was removed and that there was no histopathologic evidence of reformation of the original controlled granulomas. Though not increasing further, the fungal burdens remained at the same level as when the DXM was discontinued. This begs the question of the role of the pathogen in evading or thwarting the immune response and preventing the host from killing it. In addition to understanding what is essential for maintenance control, much more needs to be known about the progression of events at the molecular level once the immunosuppression is removed. Obviously, in a clinical situation, an antifungal agent would be given, but does this buy the host time by reducing the fungal burden to an immunologically “manageable” level, or is the host truly unable to restructure the immunity they developed following the initial infection? These questions are significant and deserve further study.

Limitations of these studies include lack of mechanistic investigations into the cellular functions within the granuloma, but they are beyond the scope of the model development and are for further study. Moreover, there were not enough animals for flow cytometry to be certain of the strength of these observations or to subject them to statistical analysis. However, they correlated well with an abundance of histopathological data. Finally, dexamathasone is a very broad, potent immunosuppressant with a multitude of immunologic targets, and it induces other metabolic changes in the animals. Studies employing immunosuppressants with more limited targets and potentially fewer systemic side effects would assist with unraveling the mechanisms of maintenance control of CM.

In conclusion, our findings provide a firm basis for future studies into the mechanisms of maintenance of host control and immunological recovery, or lack thereof in mice, following immunosuppression. It also raises questions about pathogenicity mechanisms employed by spherules when host immunity is lost. Significant inroads have been made toward understanding controlled and progressive granuloma immune mechanisms in human mycobacterial diseases [22,23,24]. With the mouse model introduced here, similar advances are now possible to better understand the control of CM.

## Data Availability

Data are available by contacting lfshubit@arizona.edu.

## Supplementary Materials

The following supporting information can be downloaded at: https://www.mdpi.com/article/10.3390/jof8100991/s1. Table S1. Receptor targets and antibody clones for flow cytometry panel; Figure S1. Flow cytometric counts of T-cell subsets; Figure S2. Flow cytometric counts of B lineage and myeloid lineage cell subsets; Figure S3. Plasma cells in lesions of untreated and immunosuppressed mice; Figure S4. Gating scheme for CD19+ B lineage cells.
