# Supplementary material for: Reactivation of Coccidioidomycosis in a Mouse Model of Asymptomatic Controlled Disease
Source: J Fungi (Basel). 2022 Sep 21;8(10):991. doi: 10.3390/jof8100991 (PMC9605249; doi:10.3390/jof8100991)
Supplement: Supplementary file 1 [file jof-08-00991-s001.zip › jof-1894636-supplementary.pdf]

Reactivation of Coccidioidomycosis in a Mouse Model of Asymptomatic Controlled Disease

Lisa F. Shubitz, Daniel A. Powell, Sharon M. Dial, Christine D. Butkiewicz, Hien T. Trinh, Amy P. Hsu, Adam Buntzman, Jeffrey A. Frelinger and John N. Galgiani

Table S1. Receptor target and antibody clone for flow cytometry panel.

Target	Clone	Fluorochrome	Supplier	Catalog Number
CD11b	M1/70	eFluor 450	eBioscience	48-0112-82
CD11c	N418	PE-Cy7	BioLegend	117318
CD19	6D5	R-PE	BioLegend	115508
CD24	30-F1	R-PE	BioLegend	138504
CD25	PC61.5	Brilliant Violet 605	BioLegend	102036
CD3	17A2	APC-eFluor 780	eBioscience	47-0032-82
CD4	GK1.5	Alexa Fluor 700	eBioscience	56-0041-82
CD44	IM7	APC	BioLegend	103012
CD62L	MEL-14	R-PE	BioLegend	104408
CD64	X54-5/7.1	Brilliant Violet 605	BioLegend	139323
CD8a	53-6.7	APC	BioLegend	100712
IgD	11-26c	APC	eBioscience	17-5993-82
IgM	II/41	FITC	BD Pharmingen	553437
Ly6G/Ly6C	RB6-8C5	eFluor 450	eBioscience	48-5931-82
MHC-II	M5/114.15.2	APC	eBioscience	17-5321-82

Figure S1. Flow cytometric counts of T-cell subsets

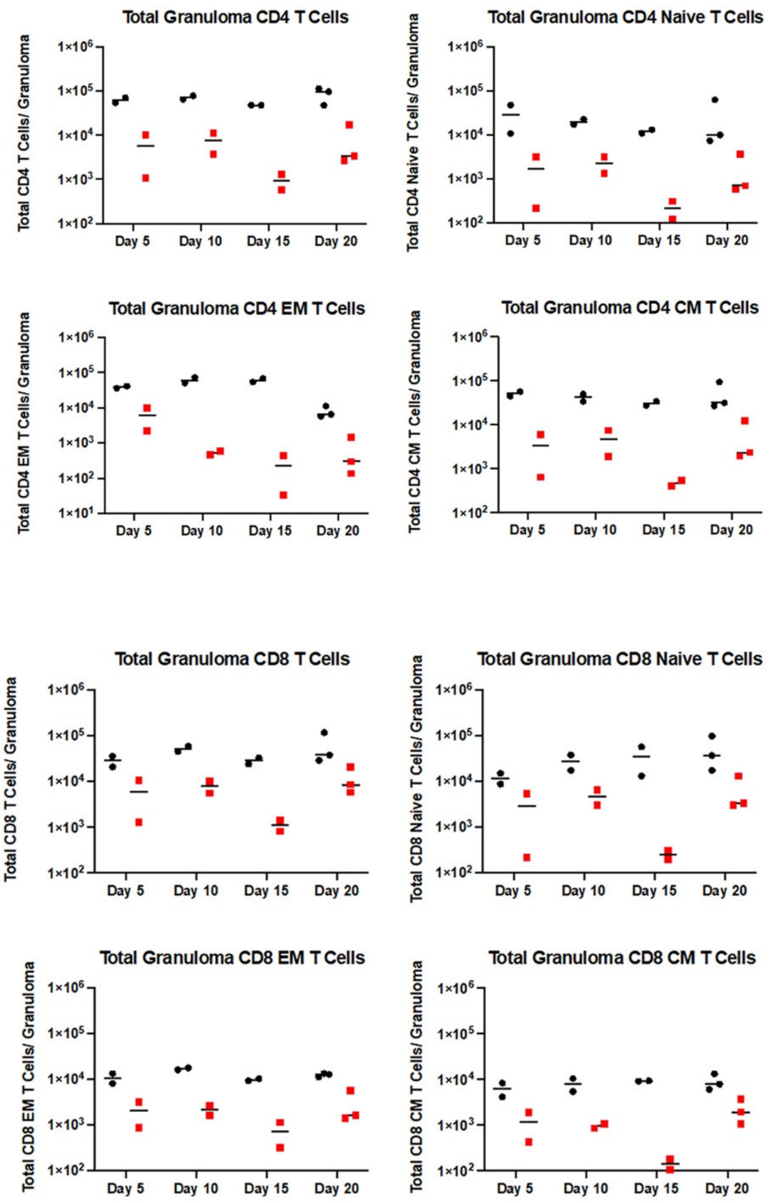

Figure S1. Flow cytometric counts of CD4 and CD8 T-cells and subsets from lung granulomas on days 5, 10, 15, and 20 after starting immunosuppressive doses of DXM. Black circles – untreated control mice; red squares – DXM treated mice, 6 mg/L in drinking water. EM= effector memory; CM= central memory

Figure S2. Flow cytometric counts of B lineage and myeloid lineage cell subsets

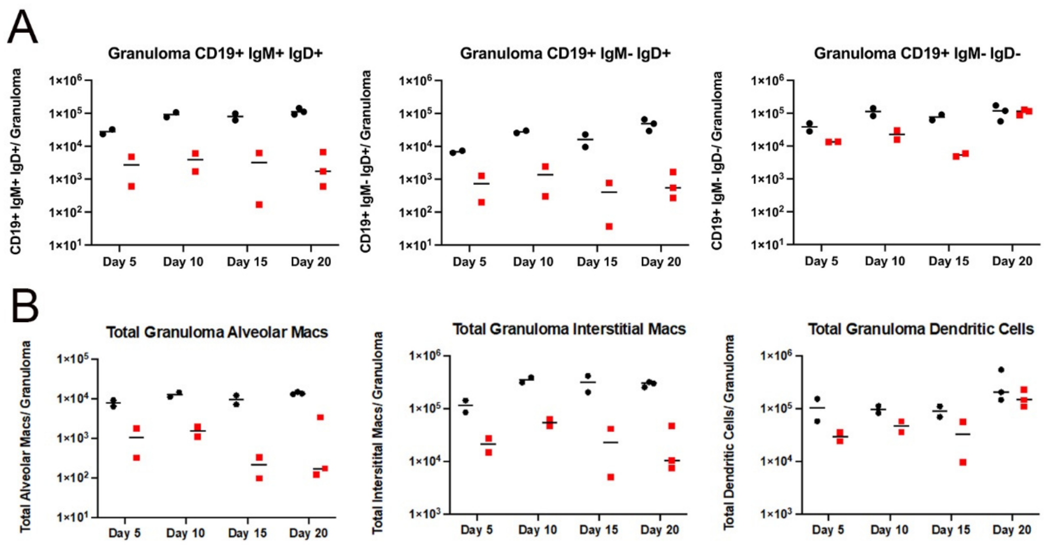

Figure S2. (A) CD19^{+} naïve (IgM^{+}IgD^{+}), maturing (IgM^{-}IgD^{+}), and mature/memory (IgM^{-}IgD^{-}) B lineage cells from lung granulomas on days 5, 10, 15, and 20 after starting immunosuppressive doses of DXM. (B) Macrophage subsets. Black circles – untreated control mice; red squares – DXM treated mice, 6 mg/L in drinking water.

Figure S3. Plasma cells in untreated and immunosuppressed granulomas

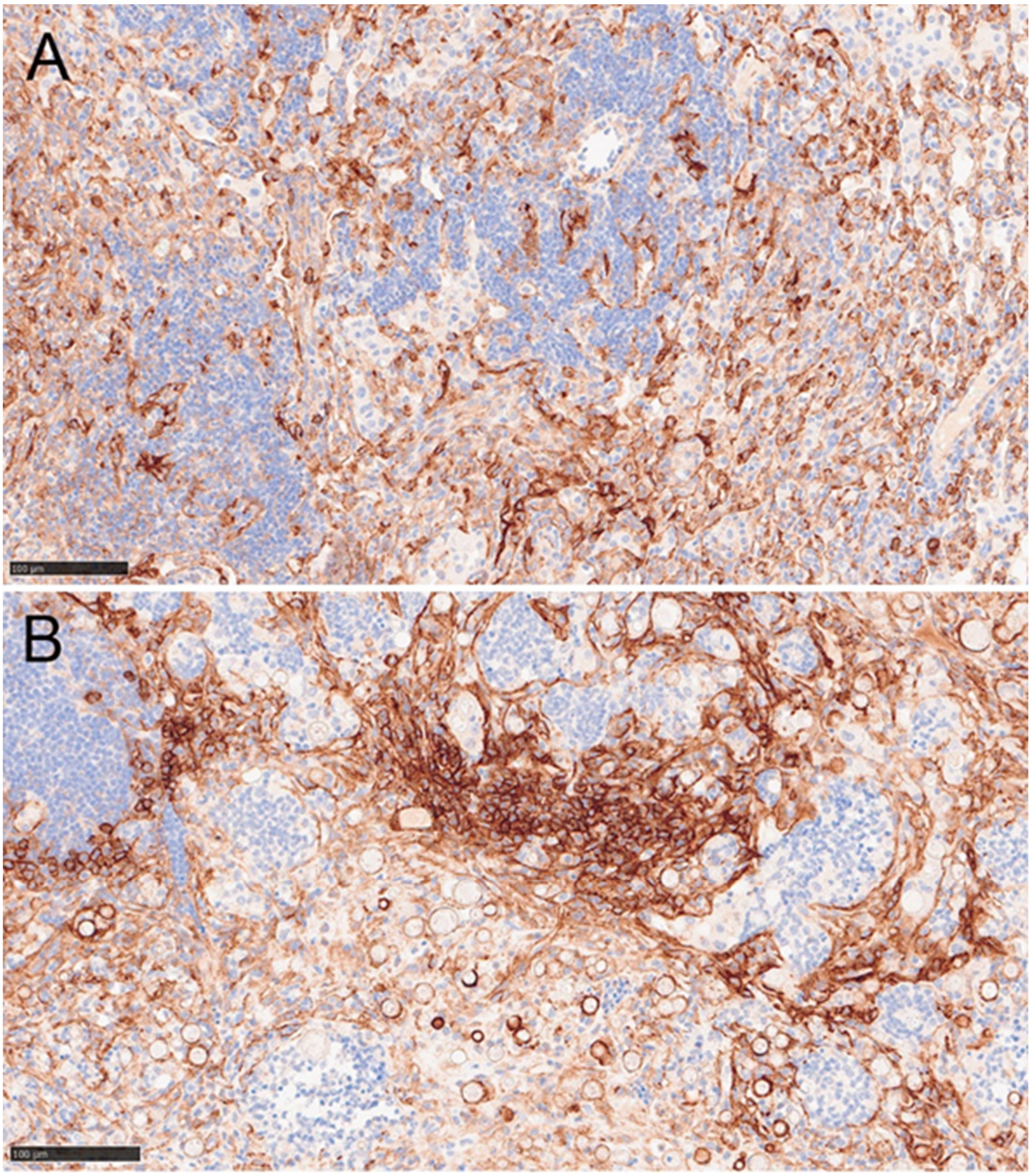

Figure S3. There are scattered plasma cells in the controlled granulomas of untreated mice (A) but by day 20 of DXM administration, sheets of plasma cells are present (B) that most likely account for the increase in CD19^{+}IGM^{-}IgD^{-} B lineage cells observed by flow cytometry. (Stain – anti-CD138 with hematoxylin counterstain; magnification x200)

Figure S4. Gating scheme for CD19$^+$ B lineage cells.

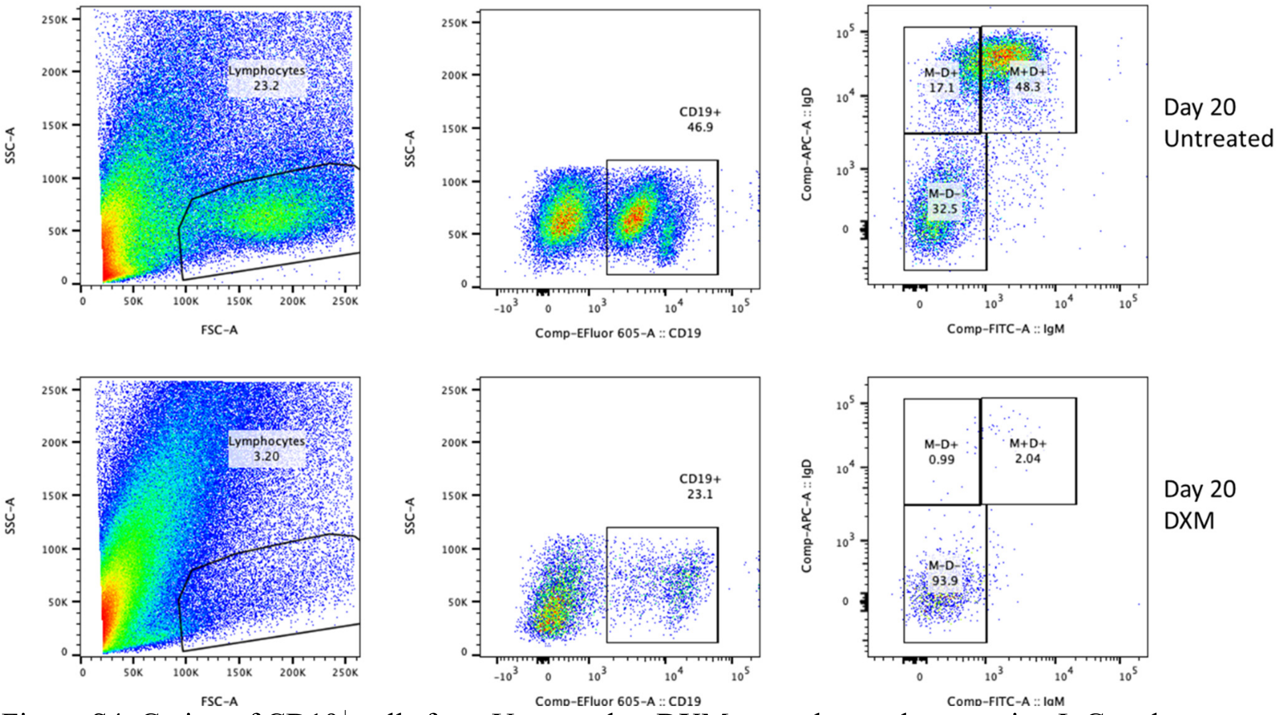

Figure S4. Gating of CD19$^+$ cells from Untreated or DXM treated granulomas using IgG and IgM antibody shows that the majority of cells in the immunosuppressed mice on day 20 (Day 20 DXM) are negative for both. This population is most characteristic of plasma cells, which were verified by histopathological and immunohistochemical observations.
